# Cognitive decline as the main manifestation of diabetic striatal disease but without involuntary movements: a case report

**DOI:** 10.1186/s12883-023-03452-8

**Published:** 2023-11-30

**Authors:** He Li, YiRan Cheng, Wei Tang, YiBin Hu, GeHui Jia, Tong Wu, KangFeng Wang

**Affiliations:** 1 Shandong University of Traditional Chinese Medicine, 250355 Jinan, China; 2 Affiliated Hospital of Shandong University of Traditional Chinese Medicine, 250014 Jinan, China; 3 Dalian University Affiliated Xinhua Hospital, Dalian, 16021 China

**Keywords:** Diabetic Striatopathy, Diabetes non ketotic hemichorea, Chorea, Subacute cognitive decline

## Abstract

Diabetic striatopathy (DS) is a rare central nervous system complication of diabetes mellitus, characterized mainly by non-ketotic hyperglycemia and lateralized involuntary movements. Patients with diabetic striatopathy manifested solely by subacute cognitive decline were rarely reported. In this paper, we report a patient with DS who presented solely with subacute cognitive decline without involuntary movements, and cranial CT showed bilateral high density in the basal ganglia. In contrast, SWI showed microhemorrhages in the right caudate nucleus head. After one week of treatment, including glycemic control, the patient showed significant improvement in cognitive function, while a repeat cranial CT showed improved hyperdensity in the right basal ganglia region. 1 month later, at telephone follow-up, the patient’s symptoms did not recur.

Diabetic striatopathy is a rare complication of the central nervous system in diabetes. DS is mainly manifested by non-ketotic hyperglycemia and unilateral involuntary movement, also known as hemichorea associated with non-ketotic hyperglycemia (HC-NH) [1]. DS with subacute cognitive impairment as the main symptom but no involuntary movement is extremely rare in clinical practice. This article reports the clinical manifestation, diagnosis, treatment process, follow-up results, and other relatively complete clinical processes of a patient with DS whose main manifestation is only subacute cognitive decline. The purpose is to introduce the diagnosis and differential diagnosis process of DS with subacute cognitive decline and high-density lesions in the bilateral lenticular nucleus to clinicians, to increase the understanding of neurologists about this type of DS, and improve the efficiency of diagnosis and treatment.

## Case report

A 73-year-old male patient with associate degree suffered delayed response, memory loss slurred speech. and increased length and frequency of sleep, but generally conscious state with no disturbance of consciousness a week before admission to the Neurology Department of Affiliated Hospital of Shandong University of Traditional Chinese Medicine. These symptoms occurred with no significant inducement. The patient had a 20-year medical history of type-2 diabetes(T2D), he took glimepiride and acarbose to control blood glucose, but the effect was poor. The patient also had a history of hyperlipidemia. The patient denied any food or medication allergy in the past.

Neurological examination revealed impaired consciousness, slurred speech, memory decline, and calculation decrement, while his time orientation, location orientation, object naming, language repetition and long-term memory were normal. Examination of cranial nerves showed no abnormalities. His myodynamia, tendon reflex, muscle tone of four limbs, superficial sensation and deep sensation were normal; Babinski’s sign was negative bilaterally. Assessment of daily living ability(ADL), Mini-Mental State Examination (MMSE), and Montreal Cognitive Assessment Test (MoCA) showed abnormalities in cognitive functioning. His ADL score was 75/100, MMSE score was 18/30, and MoCA score was 16/30.

**Fig. 1:**
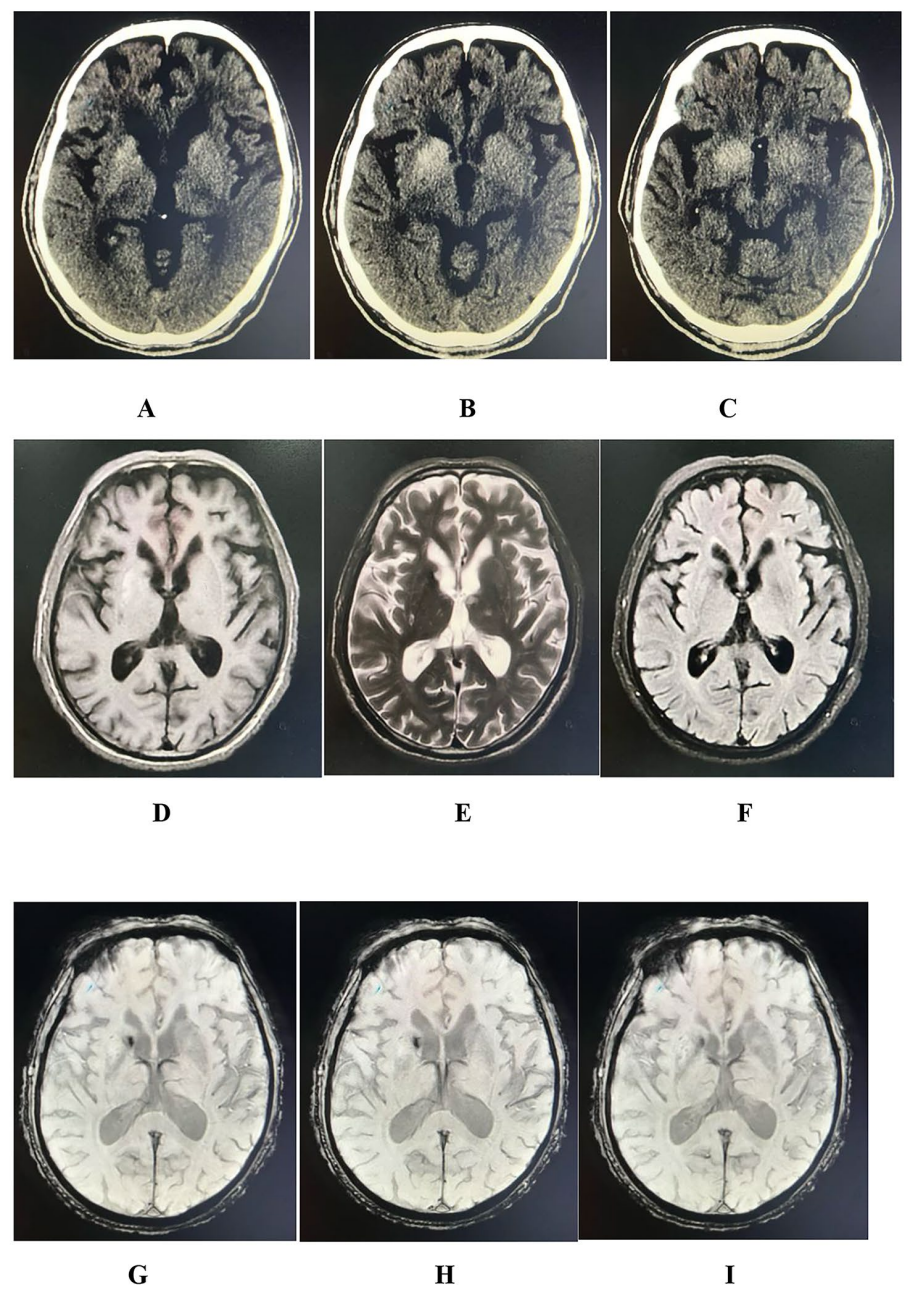
The patient’s imaging results. **(A-C)** Brain CT showed high density lesions in both basal ganglia regions, with more severe lesions on the right. **(D-F)** Brain MRI shows abnormal signals at the right head of caudate nucleus, with T1 showing high signal, T2 and Flair showing low signal. (G-H) SWI shows low signal at the head of the right caudate nucleus

After admission, the patient received brain CT, brain MRI, and brain SWI to examine his brain structure; he also received several blood tests to measure blood glucose and serum lipids, Glycosylated hemoglobin, and other blood indices. Brain CT suggested high-density lesions in the bilateral lateral caudate nucleus and the region of the nucleus accumbens. Brain MRI - T1 showed high signal-intensity lesions in the right caudate and lenticular nuclei, while T2 showed low signal-intensity lesions in the corresponding regions. SWI indicated the patient had a low signal lesion in the right caudate nucleus (Fig. 1). Blood tests showed hyperglycemia up to 12.13 mmol/L (Random fasting blood glucose level) with strong positive uric sugar (+++) and Glycosylated hemoglobin of 13.8%. The tests also revealed hyperlipidemia with triglyceride up to 1.86mmol/L, Total cholesterol up to 9.14 mmol/L, and Low-density lipoprotein cholesterol up to 6.23mmol/L. Other tests, such as serum homocysteine, blood coagulation and routine blood test showed no abnormal results.

**Fig. 2:**
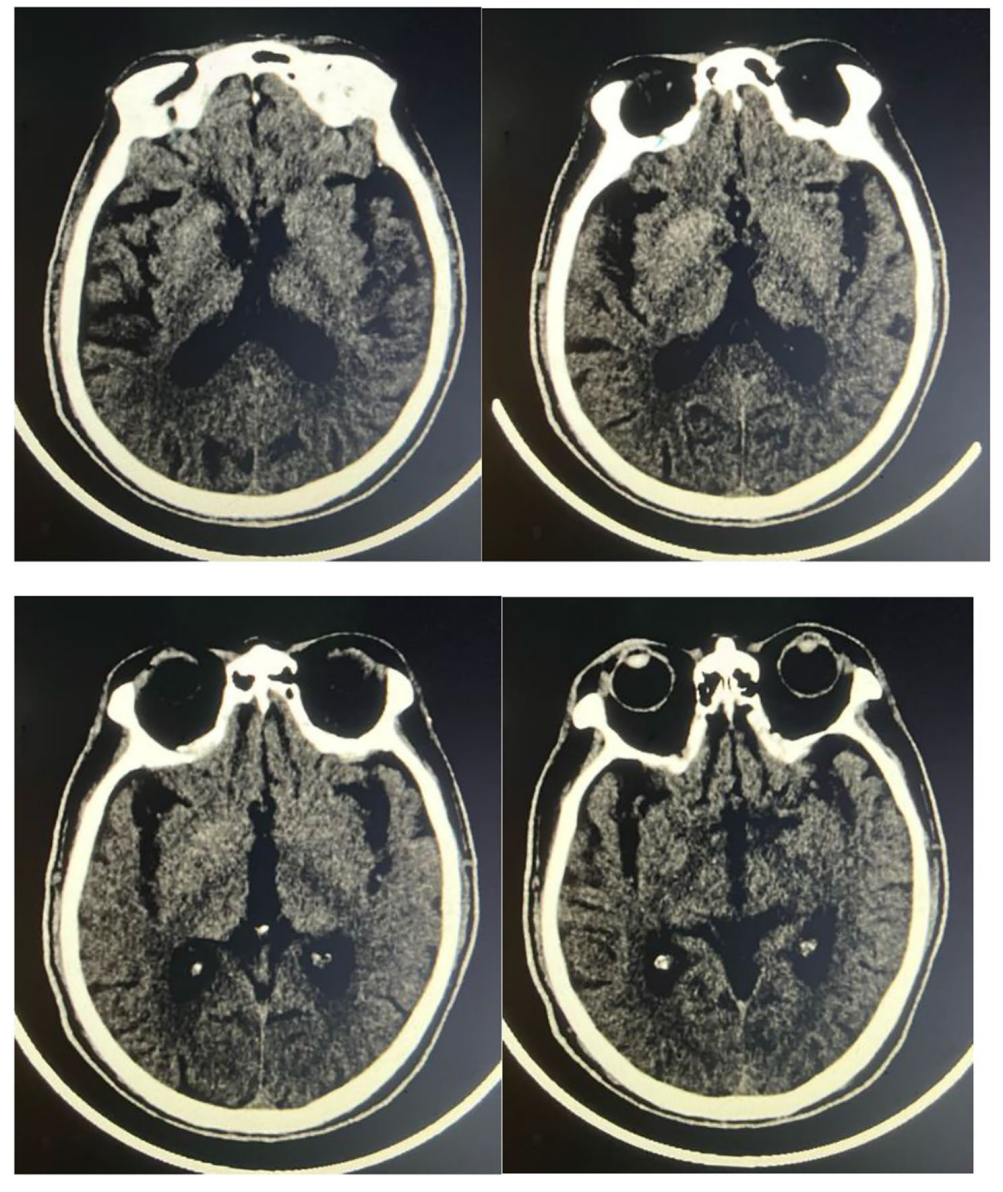
The patient’s imaging results. Brain CT showed a significant decrease in CT values of high-density lesions in both basal ganglia regions compared to previous studies

According to the patient’s hyperglycemia, he received a low-carbohydrate diet, once-daily insulin degludec injection(long-acting insulin, 14 U) before sleep, glimepiride 2 mg per day and acarbose 50 mg with every meal. Due to hyperlipidemia, he received atorvastatin calcium tablets 20 mg daily. Since admission, the patient’s blood pressure has been maintained at 130–140/80-85mmHg, so he did not reveive any antihypertensive drugs. The patient’s blood glucose level returned to normal on the third day of his treatment. Interestingly, this patient’s cognitive function improved significantly after seven days of treatment. He can communicate with others more smoothly, speaking more fluently, and his reaction retardation also improved. However, the patient still declined in recent memory and computational power. His ADL score turned 85/100, MMSE score turned 24/30, and MoCA score turned 22/30. We re-examined the patient’s brain CT. CT showed that the high-density lesions in the right caudate and lenticular nuclei of the patient were significantly smaller than before (Fig. 2). The patient was discharged due to improvement. We conducted a telephone follow-up of the patient one month later. Unfortunately, according to the patient’s family, his blood glucose control was still poor, with fasting blood glucose of 12–13 mmol/L, and his cognitive function significantly decreased compared to when he was discharged.

## Discussion

This patient mainly had the manifestation of a subacute decline in cognitive function, while brain CT revealed high-density lesions mainly in the right basal ganglia region. Due to the simultaneous detection of CT high density and T1 high signal in this patient, such manifestations could be easily misdiagnosed as cerebral hemorrhage, calcification, methanol poisoning, and other diseases. Because the patient’s high-density lesions and T1 phase high signal lesions on brain CT were confined to the striatum, his blood sugar was poorly controlled, his symptoms were relieved and the imaging manifestations improved after blood sugar control, and there was no medical history of hypertension, brain edema, or methanol exposure, so we considered this patient as DS. In addition, subacute cognitive decline should also be distinguished from vascular causes, infectious encephalitis, Creutzfeldt Jakob disease, toxic metabolic diseases, and autoimmune encephalitis (including paraneoplasia) [2].

DS is a rare disease related to poor blood glucose control. It usually shows unilateral involuntary movement and can occur in patients with T2D and type-1 diabetes(T1D) [1, 3, 4]. Acute-onset chorea is the main manifestation of DS [5], related to DS involving the new striatum. γ DS patients without involuntary movement are sporadic. Of the 176 DS patients included by Choon Bing Chua et al., only 2.3% of patients showed striatal involvement without the clinical manifestation of chorea [1]. DS patients who had previously been reported to have no involuntary movements were mainly manifested as consciousness disorders, epilepsy, limb weakness, earache, and dysphagia [6–8]. However, this kind of case, characterized by decreased cognitive function, may be rarely reported so far.

The importance of basal ganglia lesion especially caudate nucleus lesion in cognitive impairment had been confirmed by research in behavioral and basic neurology [9]. Caudate nucleus lesion can lead to significant cognitive impairment in patients [10]. This may be explained by the fact that the damage to structures such as the caudate nucleus leads to impaired integrity of the cholinergic pathway structure in the radiative coronal and basal ganglia regions and the frontal lobe-basal ganglia circuitry impairment [11, 12]. The orbitofrontal-basal ganglia circuit, including the caudate nucleus and putamen, regulates specific cognitive functions. Research showed that activation of the putamen in the basal ganglia was associated with working memory of brain, while activity levels in the head of the caudate nucleus were related to executive processes [13]. The CT scan of this patient showed that high density mainly occured in the right caudate nucleus, and the low signal lesions displayed by SWI were also mainly located in the right caudate nucleus rather than the putamen, indicating that there was relatively severe damage to the caudate nucleus. We speculated that this patient’s cognitive function decline was related to the injury to the right caudate nucleus head. Previous studies showed that the caudate nucleus received neuronal inputs from the prefrontal cortex, closely related to cognitive function [14]. When the caudate nucleus was damaged, and the cortex responsible for cognitive function was not damaged or atrophied, the destruction of the limbic system circuit still could lead to confabulation, dementia, speech defects, and neglect syndrome [15]. What’s more, when the neostriatum is injured, it can lead to increased excitability in the cortical motor area, manifesting typical involuntary movement [16]. So the patient’s relatively mild neostriatal damage may be the reason of the absence of involuntary movement.

The primary treatment for DS is to control hyperglycemia [17]. However, only a quarter of patients could achieve the goal of correcting DS-related chorea by controlling their blood glucose, and most patients still needed additional anti-chorea drugs to control their symptoms [1]. Relevant research showed that [1] the imaging changes of DS abnormalities were reversible, and some patients with CT high-density lesions could recover within ten days after active treatment. Most patients with imaging abnormalities could recover within three months. After seven days of actively controlling blood glucose and other treatments, the CT value of the patient’s brain CT lesions began to decline. At the same time, without using drugs such as cholinesterase inhibitors(CHEIs) to improve cognitive function, patients’ cognitive function improved, which suggested that DS patients only with the symptom of cognitive decline might improve their cognitive function by positive control of their blood glucose. However, a larger sample of clinical studies are required to confirm this hypothesis.

### Conclusion

Subacute cognitive decline could be the only manifestation of DS, which may be related to head of the caudate nucleus injury. In case of treatment delay, it needs careful differentiation in clinical practice to avoid misdiagnosis as cerebral haemorrhage or calcification. For DS patients only with cognitive decline, the symptoms can be relieved by positive blood glucose control rather than using drugs with cognitive improvement function such as CHEIs.

## Data Availability

The data used is available from the corresponding author upon reasonable request.

## Abbreviations

CT: Computed tomography
MRI: Magnetic resonance imaging
SWI: Susceptibility weighted imaging

## References

[1] Chua CB, Sun CK, Hsu CW (2020). Diabeticstriatopathy’: clinical- presentations, controversy, pathogenesis, treatments, and outcomes. Sci Rep.

[2] Sarah HB, Seth A, Cohen A, et al. Clinical reasoning: a 68-year-old man with rapid cognitive decline. Neurol August. 2019 93;13:315–8. 10.1212/WNL.0000000000007954 31405937

[3] Ondo WG (2011). Hyperglycemic nonketotic states and other metabolic imbalances. Handb Clin Neurol.

[4] Ottaviani S, Arecco A, Boschetti M (2022). Prevalence of diabetic striatopathy and predictive role of glycated hemoglobin level. Neurol Sci.

[5] Ryan C, Ahlskog JE, SavicaR. Hyperglycemic chorea/Ballism ascertained over 15years at a referral medicalcenter.Parkinsonism Relat Disord 2018,48(3):97–100. 10.1016/j.parkreldis.2017.12.032 29305082

[6] Sato H, Hamano M, Fushimi E (2017). Diabetic striatopathy manifesting as severe consciousness disturbance with no involuntary movements. Diabet Med.

[7] Tung C-S, Guo Y-C, Lai C-L (2010). Irreversible striatal neuroimaging abnormalities secondary to prolonged, uncontrolled Diabetes Mellitus in the setting of Progressive focal neurological symptoms. Neurol Sci.

[8] Shobha N, Sinha S, Taly A (2006). Diabetic nonketotic hyperosmolar state: interesting imaging observations in 2 patients with involuntary movements and seizures. Neurol India.

[9] Bostan AC, Strick PL. The basal ganglia and the cerebellum: nodes in an integrated network. Nat Rev Neurosci, 2 0 1 8, 1 9 (6) : 3 3 8 – 3 5 0. 10.1038/s41583-018-0002-7 PMC6503669 29643480

[10] BIESBROEK JM, WEAVER N A, BIESSELS GJ (2017). Lesion location and cognitive impact of cerebral small vessel Disease. Clin Sci (Lond).

[11] ZHU H, WANG W, LI H (2019). Basal Ganglia-Cortical Circuit Disruption in Subcortical Silent Lacunar infarcts. Front Neurol.

[12] LI Z X ZUOLJ, ZHU R Y (2018). The relationship between Cerebral White Matter Integrity and cognitive function in mild Stroke with basal Ganglia Region infarcts. Sci Rep.

[13] Knutson B, Gibbs SE (2007). Linking nucleus accumbens dopamine and blood oxygenation. Psychopharmacology.

[14] Grahn JA, Parkinson JA, Owen AM (2008). The cognitive functions of the caudate nucleus. Prog Neurobiol.

[15] ang Kwon SH (2018). Injury of the Prefrontocaudate Tract in a patient with a bilateral Caudate Infarct. Balkan Med J.

[16] Hsiao PJ, Kuo CC, Kuo TY (2019). Investigation of the relationshipbetween non-ketotic hyperglycemia and hemichorea-hemiballism:a case report. Medicine(Baltimore).

[17] Xu Y, Shi Q, Yue Y (2022). Clinical and imaging features of diabetic striatopathy: report of 6 cases and literature review. Neurol Sci.

